# Linguistic Focus Promotes the Ease of Discourse Integration Processes in Reading Comprehension: Evidence From Event-Related Potentials

**DOI:** 10.3389/fpsyg.2018.02718

**Published:** 2019-02-01

**Authors:** Chin Lung Yang, Huili Zhang, Haifeng Duan, Haihua Pan

**Affiliations:** ^1^Laboratory of Theoretical Psycholinguistics, Department of Linguistics and Modern Languages, Faculty of Arts, Chinese University of Hong Kong, Hong Kong, China; ^2^School of Foreign Studies, Southern Medical University, Guangzhou, China; ^3^Department of Minority Languages and Literatures, Minzu University of China, Beijing, China

**Keywords:** focus processing, information structure, ERPs, P200, contrastive focus, informational focus

## Abstract

Psycholinguistic studies of focus processing have yielded varying results regarding how focus affects language processing. We report the results of an event-related potential (ERP) experiment that used question-answer pairs in a discourse to manipulate whether a target word was contextually focused, contrastively focused, contextually defocused, or contextually neutral. We found a negative-going waveform that was sustained in the time-course (250–800 ms after the target word onset) with a maximum over frontal-central scalp sites. As the structure of the discourse made the target word more focused, the negative-going deflection was systematically reduced. We also observed a frontal positive-going waveform that was larger for the focus-marked words relative to the neutral target word in an earlier time window (150–250 ms, P200), which may reflect increased attention allocated to the focused items. We propose that the reduced negative ERPs for the focused words reflects facilitation of meaning integration when focus functions to establish reference in the discourse representation. This can be attributed to extra attention paid to the focus-marked items that in turn promotes the prominence of focus-marked referent and prompts the contextual priming mechanism that facilitates the access of propositionally relevant items in text memory during reading.

## Introduction

Understanding a sentence in a communicative context requires readers/listeners to make use of multiple cues to identify the information structure, i.e., the *focus* that signals the most *prominent* linguistic constituent in the sentence (Halliday, 1967) as compared to information that is presented as background. For instance, John can be signaled as the focus via prosodic contour, such as *JOHN talked to Mary*, or by syntactic structure, such as *It was John that talked to Mary*, where the meaning presupposing that someone talked to Mary is presented as the background knowledge while John is presented as the newly asserted information, i.e., as “the one” who talked to Mary.

Linguistic focus may be realized in various functionally distinctive manners (Zimmermann and Onea, 2011). For instance, Mary in (1d), when preceded by the question (1b), presents given information, whereas both Mary in (1d) and Jennifer in (1e), when in response to the questions (1a) and (1b), respectively, mark new and focused information (e.g., informational focus, also discourse newness). The word *Jennifer*, in fact, is *contrastively* focused because such a focus is to express corrective or exhaustive identification of its referents (i.e., It was Jennifer instead of Mary who was kissed by John). These referents are assigned *narrow focus*—one that is made by virtue of specified contextual information—which is in contrast with the circumstance where no specified information constraint is given—i.e., the question (1c) and its answer, which could be either (1d) or (1e). In such cases, a *wide/broad focus* reading is computed and the entire sentence, namely (1d) and (1e), would be interpreted as new information (Cinque, 1993).

1. Speaker A: a. Whom did John kiss?
    b. Did John kiss Mary?
    c. What happened?
Speaker B: d. John kissed Mary.
    e. John kissed Jennifer.

Different types of focus have been assumed to be associated with different underlying mechanisms during the comprehension processes (Benatar and Clifton, 2014). Compared to given information entailed by the discourse context, new (and focused) information would require a change in the discourse representation, e.g., introducing a new referent (Benatar and Clifton, 2014; also Burkhardt, 2006). For the contrastive focus that entails a new relation, additional processes are required to revise and update the representation that contains the entities involved (Benatar and Clifton, 2014). Wide focus presents a special case relative to narrow focus. Because the whole sentence is presented as new information (Cinque, 1993), considerable processes would be required to establish its overall meaning. Indeed, eye-tracking studies reported that increased linguistic focus is associated with longer reading times, suggesting costly processing for deeper encoding of the focused word (Lowder and Gordon, 2015) and when the discourse content requires updating or revision of readers' discourse representation (Benatar and Clifton, 2014). Benatar and Clifton (2014), across experiments, reported graded effects of discourse focus processing, with given materials being read faster than materials that are simply new (thus focus-marked, Experiments 1 and 2), which are still being read faster than materials that require changes to the discourse model (i.e., contrastive focus, Experiment 3). Lowder and Gordon (2015) compared the processing of syntactic focus varying by degrees (e.g., focused, neutral, defocused) in a single experiment, and reported longer times for increased linguistic focus, with the focused word being processed slower than the neutral word, which is in turn being processed still slower than the defocused word.

While these studies provide some evidence for costs when processing focused material (see also Birch and Rayner, 1997; Price and Sanford, 2012), there is also evidence that focus promotes the ease of integrating a word with the context meaning—focused entities were processed more quickly than non-focused ones (e.g., Morris and Folk, 1998; Birch and Rayner, 2010; Chen et al., 2012, re-reading times), and that focus exerts no effect on reading speed (Ward and Sturt, 2007). A similar discrepancy was observed in electrophysiological studies as well. Event-related potential (ERP) studies of focus processing have yielded varying effects that appear to depend on the setup of focus assignment (syntactically-/discourse-assigned focus), and the exact processes being compared in experiments—e.g., context-congruent vs. context-incongruent focus, focused vs. less-/non-focused (Stolterfoht et al., 2007). For auditory comprehension, both Johnson et al. (2003) and Hruska et al. (2000) reported that the processing of focused constituents, relative to non-focused (given) material, evoked a late positivity. In reading comprehension, Cowles et al. (2007) reported a right lateralized negativity of 200–500 ms when a word occurring in the focus position of it-cleft sentences mismatched with the context-specified anticipated focus. Bornkessel et al. (2003) reported a parietal positivity (280–480 ms) associated with the processing of focus constituents. Stolterfoht et al. (2007) showed a bi-lateralized sustained positive shift (350–1,300 ms) associated with the revision processing of a focus structure. Chen et al. (2014) found that focused words elicited a larger late positivity (500–700 ms) than the non-focused words.

The reason for this lack of consistency, as noted by Benatar and Clifton (2014), may lie in the fact that focus has been used as a broad, multifaceted construct that reflects a collection of concepts (Buring, 2007), and there has not been a consensus regarding how different kinds of focus would be distinguished and manipulated in experiments. The inconsistent claims in previous studies may reflect distinctive cognitive mechanisms involved in the online processing of linguistic focus, depending on the particular type of manipulations (Stolterfoht et al., 2007; Lowder and Gordon, 2015). Questions about how and when different types of focus affect language processing remain to be answered. The current study addresses this issue by taking an approach that allows for an assessment of the effect of different types of focus during the comprehension processes.

One potential caveat in previous work is that most studies have mainly compared two types of focus category in the investigation, e.g., focus-congruent vs. focus-incongruent (Cowles et al., 2007), focus vs. non-focus entities (Chen et al., 2012, 2014), given vs. new entities (Benatar and Clifton, 2014, Experiment 1 and 2), contrastive vs. given foci (Benatar and Clifton, 2014, Experiment 3), utterances with/without focus markers (e.g., *only/even* in Spalek et al., 2014), sentences with exclusive *only* vs. inclusive *even* focus markers (Filik et al., 2009), and utterances of canonical vs. non-canonical types (Bornkessel et al., 2003; Stolterfoht et al., 2007). Some studies, in effect, manipulated focus by placing the focused word in syntactic structures that differed from those of non-focused materials (e.g., canonical vs. non-canonical types as in Bornkessel et al., 2003; Stolterfoht et al., 2007; with/without focus markers as in Chen et al., 2014), and with specific constructions in a manner that would implicate somewhat contrastive functions (e.g., clefting in Cowles et al., 2007 as well as the use of Chinese focus marker *shi* in Chen et al., 2012, 2014). As noted by Birch and Rayner (2010), comparing the effect of focus vs. non-/less-focus by virtue of varying syntactic positions might not disentangle effects of focus from effects of syntactic prominence. Benatar and Clifton (2014), additionally, suggested that focusing a word by clefting it might incur unexpected semantic/pragmatic effects (e.g., exhaustiveness, existence presupposition, etc.) to bear on the processing, and thus must be distinguished from the informational focus (Torregrossa, 2012). In the experiment reported here, we manipulated a *range* of focus conditions within a single experiment and used the fine-grained, timing-sensitive online measure of neural processing (ERPs) to expose their relative prominence and real-time interactions. In particular, we implemented a design that controlled the potential confounds (shown below). These, together, would allow us to examine more closely how different types of focus affect real-time informationally-based processes.

We presented passages like the one in Table 1 that used question-answer pairs to manipulate contextually focused information in the investigation. The first sentence of each passage introduces three discourse referents, all occurring in the subject position, and sets up the context for the following question-answer pair sentences. The context sentence describes an event scenario that implicates unbiased involvement of three discourse referents. For instance, as shown in Table 1, the verb phrase in the context sentence of Set 1 *dàdǎchushou* (“fight one another”) denotes three male characters fighting with one another, and the following question (the second sentence), through the manipulations of *wh*-question formats (i.e., *whom*-question, *who*-question, and *what*-question), sets up the focus status of the target word (i.e., 周兵 *Zhoubing*, the grammatical object with the patient role) in the answer (the third sentence). The verbs in the answer sentence are generally monotransitive. Some verbs are ditransitive, like *y*í*ng* (“defeated”) in the sample stimuli Set 3, but the monotransitive meaning is acceptable on the basis of linguistic context we created. ERPs time-locked to the target words were extracted and examined. In the Focused condition, the target word was made discourse-prominent by virtue of the preceding *whom*-question that placed focus on the grammatical object (also patient) of the answer sentence. In the Contrastive condition, the target word in the answer sentence was to correct what was asserted in the preceding question (i.e., it is not 子健 *Zǐjiàn* but 周兵 *Zhōub*ī*ng* that 國強 *Guòqiáng* beat). In the Defocused condition, the target word was discourse-deemphasized by the preceding *who*-question that placed focus on a different region of the answer sentence (i.e., the grammatical subject with the agent role). In the Neutral condition, which also served as a baseline, the target word in the answer sentence received no specific focus status (i.e., the wide focus) because the entire sentence constitutes the answer to the preceding *what happened* question (Cinque, 1993). Across the four conditions, the answer sentence was identical, and the target word was always the same in all conditions and in the same grammatical object/patient position of the sentence, preceded by the same subject-verb and followed by the same complement phrase. In this way, focus manipulations in the answer involved neither varying syntactic structures nor specific constructions, which made it unlikely that the effects found in our data might be confounded with factors noted by Benatar and Clifton (2014) and Birch and Rayner (2010).

**Table 1:** Sample passages of the experiment.

**Stimuli set**	**Condition (Informational Status)**	**Context setup sentence (the 1st sentence)**	**Question-answer pair sentences**	
			**Question (the 2nd sentence)**	**Answer (the 3rd sentence)**
1	Focused	國強, 周兵, 子健在學校大打出手。 Guóqiáng, Zhōubīng, Zǐjiàn zài xuéxiào dàdǎchushǒu. Guoqiang, Zhoubing, Zijian in school fight one another. “Guoqiang, Zhoubing and Zijian were fighting one another in the school.”	國強打了誰? Guóqiáng dǎ-le shéi Guoqiang beat whom “Whom did Guoqiang beat?”	國強打了周兵, 但是出手不重。 Guóqiáng dǎ-le Zhōubīng, dànshì chūshǒu bú zhòng Guoqiang beat Zhoubing, but not heavy “Guoqiang beat Zhoubing, but not heavily.”
	Contrastive (focus)		國強打了子健 Guóqiáng dǎ-le Zǐjiàn Guoqiang beat Zijian “Did Guoqiang beat Zijian?”	
	Defocused		誰打了周兵? Shéi dǎ-le Zhoūbīng Who beat Zhoubing “Who beat Zhoubing?”	
	Neutral		發生了什麼事? Fāshēng-le shénme shì Happened what thing “What happened?”	
2	Focused	子豪,學武,仁傑在辦公室裡發生了口角。 Zǐháo, Xuéwǔ, Rénjié zài bàngōngshì lǐ fāshēng-le kǒujué. “Zihao, Xuewu and Renjie quarreled in the office.”	子豪罵了誰? Zǐháo mà-le shéi? “Whom did Zihao scold?”	子豪罵了學武, 但是情有可原。 Zǐháo mà-le Xuéwǔ, dànshì qíngyǒukěyuán. “Zihao scolded Xuewu, but this was excusable.”
3	Focused	文博, 楊軍, 趙傑在田徑場比賽長跑。 Wénbó, Yángjūn, Zhàojié zài tiánjìngchǎng bǐsài chángpǎo. “Wenbo, Yangjun and Zhaojie were contesting a long-distance race in the athletic field.”	文博贏了誰? Wénbó yíng-le shéi? “Whom did Wenbo defeat?”	文博贏了趙傑, 但是贏得不痛快。 Wénbó yíng-le Zhàojié, dànshì yíng-de bú tòngkuai. “Wenbo defeated Zhaojie, but not happily.”
4	Focused	江順, 小陽, 高越在公園裡玩捉迷藏。 Jiāngshùn, Xiǎoyáng, Gāoyuè zài gōngyuán lǐ wán zhuōmícáng. “Jiangshun, Xiaoyang and Gaoyue were playing hide-and-seek in the park.”	小陽捉了誰? Xiǎoyáng zhuō-le shéi? “Whom did Xiaoyang catch?”	小陽捉了江順, 然後趕緊藏起來了。 Xiǎoyáng zhuō-le Jiāngshùn, ránhòu gǎnjǐn cángqǐlai-le. “Xiaoyang caught Jiangshun, and then hid quickly.”
5	Focused	清紹, 孔維, 趙琪在愚人節那天互相捉弄。 Qīngshào, Kǒngwéi, Zhàoqí zài Yúrénjié nàtiān hùxiāng zhuōnòng. “Qingshao, Kongwei and Zhaoqi teased each other on April Fools' Day.”	孔維耍了誰? Kǒngwéi shuǎ-le shéi? “Whom did Kongwei tease?”	孔維耍了清紹, 得意得不得了。 Kǒngwéi shuǎ-le Qīngshào, déyì-de bùdéliǎo. “Kongwei teased Qingshao, and was extremely pleased.”
6	Focused	廖倩,靜怡, 許姍在公司會議上互相批評。 Liàoqiàn, Jìngyí, Xǔshān zài gōngsī huìyì shàng hùxiāng pīpíng. “Liaoqian, Jingyi and Xushan criticized each other at the company meeting.”	許姍批了誰? Xǔshān pī-le shéi? “Whom did Xushan criticize?”	許姍批了廖倩, 說她總是擅自作主。 Xǔshān pī-le Liàoqiàn, shuō tā zǒngshì shànzì zuòzhǔ. “Xushan criticized Liaoqian for making unauthorized decisions.”
7	Focused	陳剛, 杜峰, 媛媛在辦公室互相誇獎。 Chéngāng, Dùfēng, Yuányuán zài bàngōngshì hùxiāng kuājiǎng. “Chengang, Dufeng and Yuanyuan praised each other in the office.”	媛媛誇了誰? Yuányuán kuā-le shéi? “Whom did Yuanyuan praise?”	媛媛誇了陳剛, 說他的業績最好。 Yuányuán kuā-le Chéngāng, shuō tāde yèjì zuì hǎo. “Yuanyuan praised Chengang for his outstanding performance.”
8	Focused	玉萱, 浩然, 韋健在公園裡玩抓人遊戲。 Yùxuān, Hàorán, Wéijiàn zài gōngyuán lǐ wán zhuā rén yóuxì. “Yuxuan, Haoran and Weijian played catch-me game in the park.”	韋健抓了誰? Wéijiàn zhuā-le shéi? “Whom did Weijian catch?”	韋健抓了玉萱, 然後就不玩了。 Wéijiàn zhuā-le Yùxuān, ránhòu jiù bù wán-le. “Weijian caught Yuxuan, and then stopped playing.”

*Note that only Set 1 shows all four levels of experimental factor, Informational Status, whereas Sets 2-8 show only the Focused condition for demonstration. As shown in Set 1, the answer (the third sentence) serves as the target sentence which is identical for all conditions. The ERPs were extracted from the onset of the target word, 周兵 Zhōubīng underlined for demonstration, of the answer (the third sentence). Likewise, target words in Sets 2-8 also are underlined. Target words in the experiment were not underlined and had the same font format and size as the other words*.

While varying ERP effects have been reported in previous work, subjected to the processes at work, and those processes being compared (Stolterfoht et al., 2007), most relevant to the present study is the established link between attention and information structure and the conclusion that focus would evoke semantic/pragmatic consequences in updating and establishing reference (Rooth, 1992). A handful of behavioral studies have demonstrated that linguistic focus tends to attract extra attention and involves more elaborate processing than non-focused elements (Cutler and Fodor, 1979; Blutner and Sommer, 1988; Sturt et al., 2004). Notably, for the type of question-answer manipulation in the present study, focused materials tended to receive more attention than non-focused ones (Benatar and Clifton, 2014). Previous studies have reported an ERP effect of ~200 ms (Klin et al., 2004; Sanford et al., 2006; Chen et al., 2012) which might be linked to the P200 component that indexed early processing of attention allocation on the focused materials (Morris and Folk, 1998; Sanford et al., 2009; Chen et al., 2012). In this context, we take the P200 to be indicative of attentional processes, with greater positivity for the focus-marked target words relative to the neutral target words.

Furthermore, according to Hamblin (1973), the semantics of a *wh*-question gives rise to the derivation of a set of propositions that are “good” answers to the question. When focus was assigned by treating the answers to a *wh*-question as focused, its function would be to resolve the target referent from a set of alternative and possibly true candidates (Karttunen, 1977) evoked by the meaning of the question that licensed focus-markings (Rooth, 1992). In this context, the N400 and the frontally-distributed sustained negativity (i.e., the referentially induced frontal negativity, Nref, in Van Berkum et al., 2007) are specific targeted components. The N400 component is a negative voltage shift that peaks between 300 and 500 ms after the onset of a word (Kutas and Hillyard, 1980). Modulations of the N400 have been found to be sensitive to meaning integration in text processing (Van Berkum et al., 2003), and focus processing (Cowles et al., 2007; Chen et al., 2014), with greater negative amplitudes associated with poorer semantic fit (Federmeier and Kutas, 2001). Thus, we take the N400 to be an indicator of semantic congruence, with greater negativity associated with the lack of ease of integrating a word with context meaning. The Nref is a sustained, negative shift with a strongly frontal distribution that was sensitive to the processing of resolving referential ambiguity, with greater amplitudes to entail costly processes to track referential processing (Van Berkum et al., 1999)^1^ at the level of the situation model (Nieuwland et al., 2007a, also see Van Berkum et al., 2007; Nieuwland and Van Berkum, 2008 for more details). Similar frontal negativity was taken to reflect the memory and control processes involved in the processing of establishing reference (Yang et al., 2010). At the discourse level, we expect the N400 and the Nref to reflect processes that retrieve, update, and establish referential meaning in readers' discourse representation (i.e., the situation model). In the present study, the neutral target word was expected to elicit an enhanced negative shift because the entire answer sentence would be processed as new information (Cinque, 1993; Burkhardt, 2006). The ERP differences between the focus-associated conditions and the neutral condition would, thus, reflect the relative ease of integrating and establishing reference with the meaning of the discourse/text.

## Materials and Methods

### Participants

Twenty-four native Chinese Mandarin speakers were recruited. They were all right-handed with normal or corrected-to-normal vision. The data from four participants were excluded due to technical problems, leaving twenty participants for the final data analysis (aged 19-27: *M* = 23, *SD* = 4). The study was approved by the Human Research Ethics Committee for Non-Clinical Faculties of The Chinese University of Hong Kong. All participants were given informed consent prior to the start of the experimental session and were compensated with HK$180 for 2 h of participation: 30 min for electro net setup and 1.5 h for the ERP experiment.

### Materials

One hundred and forty sets of three-sentence experimental passages were constructed across four conditions that varied in the opportunities they presented for informationally-based meaning integration (Focused, Contrastive, Defocused, and Neutral focus), shown in Table 1. The construction of the experimental passages follows a stringent word order in which each sentence in the passage has a similar structure as those shown in Table 1. In particular, the syntactic structure of the answer sentence was always subject-verb-object-complement, where the verb was always adjacent to its NP arguments (i.e., the grammatical subject and object), and that the target word was always the grammatical object of the answer sentence, followed by a complement phrase. The target words were all two-character words that denoted common proper names (e.g., 子健 *Zijiàn*, 周兵 *Zhoubing*, 美欣 *Guòqiáng*, 國強 *Meixin*, 家明 *Jiaming*, etc.) and were the same in all four versions. A total of 420 different two-character proper names were used in the 140 sets of passages, with no repetition of proper names across passages. It is also noteworthy that the questions were designed not to bias the answer. The three referents mentioned in the context setup sentence were swapped for the two potential undergoers (agent and patient) in the question-answer pair sentences. Approximately one-third of the two undergoers in the question-answer sentences involved the first and second referents mentioned in the setup sentences, one-third involved the second and the third referents introduced in the setup sentences, and another one-third involved the first and third referents mentioned in the setup sentences.

#### Naturalness Norming

Forty-two new university students from the same population performed naturalness norming for the experimental passages. Each participant received one list of stimulus materials, along with 80 filler passages randomly selected from the 100 filler passages in the ERP test. Across participants, the different versions of all of experimental passages were presented equally. In each trial, participants saw only one version of each experimental passage or filler passage; after reading through each passage, they were asked to rate their first impression by responding to the question “How natural do the sentences go together?” on a five-point scale (1 = very unnatural, 5 = very natural). Two participants were excluded due to excessive careless responses, leaving forty participants for the data analysis. The results indicated that the four experimental conditions were rated equally natural, *F*_(3, 117)_ = 1.25, *p* = 0.29, despite some difference in the numerical values: mean ratings for Focused: 4.1 (*SD* = 0.5), Contrastive: 3.9 (*SD* = 0.7), Defocused: 3.9 (*SD* = 0.6), and Neutral: 4.0 (*SD* = 0.6).

### Design and Procedure

The four versions of the 140 experimental passages were counterbalanced across four lists by a Latin-square design so that each participant saw only one version of each item, and each participant saw the same number of passages from each of the four conditions (i.e., 35 passages for each condition in the list). Each list also contained 100 filler passages that varied in length, structure, and content to increase the variations of the passages read for comprehension. The ERP experiment had 140 experimental and 100 filler passages that consisted of two blocks, each having equal numbers of experimental passages and filler passages. Participants were tested through two blocks, with a break of 5–10 min between blocks. The presentation of the four experimental conditions and fillers in each block was randomly intermixed, and the sequence of the two blocks was randomly assigned to each participant. This design and setup made it unlikely that participants' responses to the target word would be biased toward predicting the pattern of the experiment on the basis of the discourse structure and content of the experimental stimuli. Before the experiment, participants performed eight practice passages.

Participants initiated the trial by pressing the space bar, and the first context-setup sentence was presented all at once on the 17-inch LCD monitor screen. Participants read the context-setup sentence silently and pressed the space bar when they were ready to read the subsequent question-answer sentences. Words in the question-answer pair sentences, after a center-positioned fixation crosshair (duration 500 ms), appeared one at a time in the center of a 2 cm height × 4 cm width area of white text on a black background. Stimulus duration was 300 ms with a stimulus-onset asynchrony (SOA) of 600 ms, which was within a typical range to present the target sentence in RSVP format for ERP studies of high-order language processing across different languages. For instance, Cowles et al. (2007) and Stolterfoht et al. (2007) used SOA of 500 ms and 550–650 ms, respectively, when studying focus processing in English and German correspondingly. Likewise, in Chinese, Yang et al. (2010) and Chen et al. (2014) used SOA of 700 ms to study the processing of Chinese relative clauses and focus processing, respectively. Chen et al. (2017) used SOA of 700 ms when studying Chinese word-to-text integration processes. Hsu et al. (2014) adopted SOA of 600 ms when studying the processing of classifier in Chinese. Hung and Schumacher (2012) used SOA of 550 ms when examining the position-specific effect during Chinese discourse comprehension. A true-or-false comprehension question based on the meaning of the passage appeared for approximately half of the trials on a random basis. Their purpose was to encourage participants to read the overall passage meaning for comprehension, and immediate feedback about response accuracy was displayed on the screen following the responses. Approximately half of the comprehension questions were based on the experimental items, and half were based on the filler items. To avoid predictable responses, the comprehension questions involved slight variations in the number of items across the experimental conditions, i.e., 3–9 items per condition. The numbers of true and false responses for each item (i.e., experimental and filler) were also equally balanced. To reduce recording artifacts, participants were instructed to avoid movements and blinks as much as possible while reading throughout the question-answer sentences, but they were told that they could rest before initiating the next trial. E-prime (Psychology Software Incorporation, Pittsburgh, Pennsylvania) was used to control the experiment.

### ERP Recording and Pre-processing

The electroencephalogram (EEG) was recorded using the 128-electrode Electrical Geodesics 300 system (Electrical Geodesics Inc., Eugene, Oregon). Throughout a recording, all impedances were kept below a threshold of 40 kΩ, an acceptable level for the electrodes and amplifier used (Tucker, 1993; Ferree et al., 2001). Impedances were checked and maintained during the break between blocks to the end of recording. Six eye channels monitored eye movement and blinks. The EEG signals were digitally sampled at a rate of 500 Hz, referenced to the vertex (Cz), and hardware filtered during recording between 0.1 and 200 Hz.

After the recording session, EEG data were filtered through a low-pass finite impulse filter of 30 Hz and then segmented into 1,200 ms epochs spanning 200 ms pre-stimulus to 1,000 ms post-stimulus for the target word to examine the ERP patterns. Data were digitally screened for artifacts (i.e., ocular and muscular movements, or transient electronic artifact) and contaminated trials were removed. Ocular artifact detection was implemented by a NetStation waveform tool that used the superior right eye channel to detect and regress out eye-blinks, and the right outer canthi channel to detect and regress out eye-movements (Gratton et al., 1983). Channels that had activity of ±200 μV on more than 20% of trials were considered too noisy for use and automatically removed. Contaminated trials were removed based on following criteria: first, more than 12 channels marked noisy for a given trial using the previous noisy channel thresholding step; second, the detection of blinks through examination of superior and inferior eye channels (excepting for the right superior eye channel removed during ocular artifact detection) for voltage fluctuations of ±140 μV; and third, the detection of horizontal eye movements (e.g., saccades) through inspection of the left outer canthi electrode for voltage fluctuations of ±55 μV. Overall, a total of 7.7% trials was discarded due to artifacts. For the retained dataset, removed channels were replaced by data from neighboring channels using spherical spline interpolation (Ferree, 2006). The data were then re-referenced to the average of the channels and corrected for the Polar Average Referencing Effect (PARE; Junghöfer et al., 1999) to reduce the bias caused by the possible uneven distribution of EEG sensors and were then baseline-corrected to the average activity during a 200 ms pre-stimulus period. Average referencing is commonly used with dense-array electrode nets and does not suffer from topographical issues seen when using average references with sparse electrode nets (Dien, 1998; Luck, 2013). Crucially, the average referencing, when being used with sufficiently dense coverage over the surface of the head, has been acknowledged as a good approximation to ideal reference (Dien, 1998; Nunez, 2010) and is more psychometrically reliable than other arbitrary recording reference channels (Gudmundsson et al., 2007).

The data were then averaged for each participant for each condition. The grand average ERPs for each condition were produced by averaging the subject-averaged ERPs across subjects. To increase the accuracy and reliability of the average-reference derivation, the spatial topography was assessed by clustering the electrodes into 13 spatial regions where the mean voltage amplitudes of the channels within each region were averaged (Dien and Santuzzi, 2005). The clustered regions included three lateral (left, midline, and right) and five lobe sites (prefrontal, frontal, central, posterior, and temporal) corresponding to the international 10/20 system: midline electrodes: Fz, Cz, and Pz; lateral electrodes: F7–F8, F3–F4, C3–C4, P3–P4, T3–T4. These clusters cover a large region of the scalp where previous studies have found reliable ERP effects.

## Results

### Comprehension Probes

The mean response times and mean accuracies for each condition were the following: Focused: 2,400 ms (*SD* = 585 ms), 94% (*SD* = 6%); Contrastive: 2,497 ms (*SD* = 757 ms), 93% (*SD* = 7%); Defocused: 2,336 ms (*SD* = 611 ms), 93% (*SD* = 7%); and Neutral: 2,362 ms (*SD* = 558 ms), 94% (*SD* = 6%). The overall mean accuracy across all conditions was 94%, and this suggests that participants were reading stimuli attentively. The mean response times to the comprehension questions suggested ~100 ms response-time disadvantage for the focused items (both focused and contrastive) over the non-focused items (both defocused and neutral). This effect, however, was not reliable. The repeated measures ANOVAs found no significance for response times (both *F*_1_and *F*_2_ < 2) and for accuracies (both *F*_1_and *F*_2_ < 1). This suggests that the processing related to readers' knowledge representation of the comprehension results was not sensitive to our focus manipulations.

### ERPs

#### Visual Inspection of ERPs

**Figure 1:**
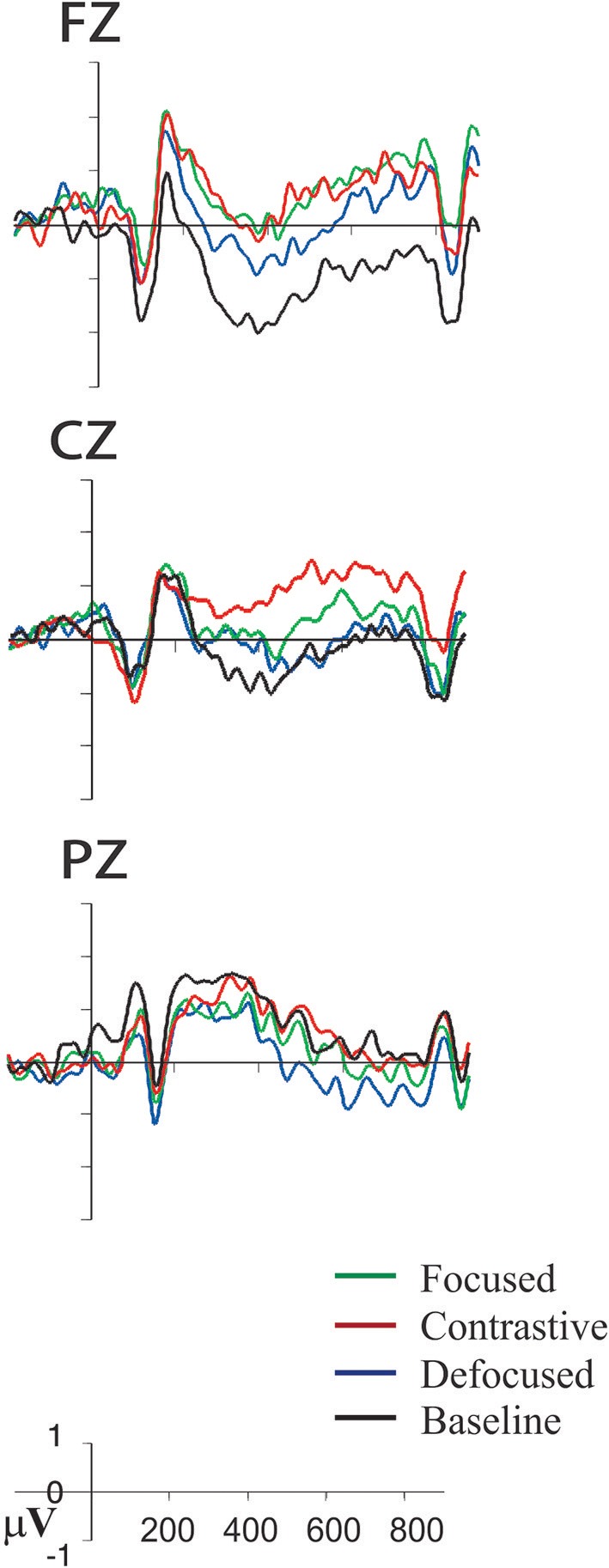
Grand average ERPs elicited by target words as a function of experimental conditions plotted for the midline electrode clusters (Fz, Cz, Pz). On the y-axis, positive amplitude values in μV are plotted upward.

Figure 1 highlights the grand average ERPs elicited by the target words in the experimental conditions. Most saliently at the frontal and central sites, the major divergence among conditions was manifested, starting at ~250 ms post-onset of the target word (shown at the Cz), in a negative-going deflection which was also sustained in the time-course for a few hundred milliseconds. Visually, the overall pattern of condition divergence remained largely similar over this sustained time course, with the Neutral condition eliciting the most enhanced amplitudes in the negative-going waveform and the Contrastive condition showing the relatively less amplitudes in the negative-going waveform, while other conditions were in between. This suggests that our experimental manipulation yielded a relatively extended negative ERP effect, similar to the effects of frontal negativity that were linked to the processing of referential ambiguity (see Van Berkum et al., 2007) and reference establishment (Yang et al., 2010). This effect differed qualitatively from Bornkessel et al. (2003) and Stolterfoht et al. (2007), where the ERP waveform for both the focus and the non-/less-focus conditions was deflected toward a *positive*-going direction, with the major divergence between the critical focus condition and the non-/less-focus condition starting at ~300 ms in Figures 1, 2 in Bornkessel et al. (2003) and ~350 ms in Figure 1 in Stolterfoht et al. (2007). Both studies indicated that the focus condition, relative to the non-/less-focus condition, elicited greater amplitudes in the positive-going waveform. In our data, we also noted a positive ERP effect that preceded the sustained negative ERPs. This earlier positive-going waveform peaked ~200 ms and was most salient at the Fz site, with larger positive amplitudes for the focused target words (both the Contrastive and Focused conditions) relative to the Neutral condition. Note that both the early positive ERPs and the following sustained negative ERPs distributed predominantly over the frontal and the central sites whereas the parietal ERPs had a less-defined peak compared to the frontal and central ERPs.

#### Analysis of ERP Effects

To test these effects, we conducted separate repeated-measures ANOVAs on the mean voltage amplitudes extracted from the two time windows: one for early positivity (150–250 ms) and one for sustained negativity (250–800 ms)^2^. Because the prefrontal (F7-F8) and temporal (T3-T4) electrode sites do not have a “midline,” separate ANOVAs were performed for each ERP component: one tested three midline clustered regions (Fz, Cz, and Pz), and the other tested five pairs of lateralized regions (F7-F8, F3-F4, C3-C4, P3-P4, and T3-T4). The ANOVAs all used two within-subjects factors: Informational Status (Focused, Contrastive, Defocused, and Neutral) and Electrodes (three for midline ANOVA and five pairs for lateral ANOVA). The lateral ANOVA includes an additional within-subjects factor of Hemisphere (Left vs. Right). We applied the Huynh-Feldt procedure to control for potential Type-I errors due to violations of sphericity (Huynh and Feldt, 1970). The corrected *P*-values are reported and the degrees of freedom are reported with original values. Table 2 outlines the results of ANOVAs for the waveform analyses of each time window. For *post-hoc* pairwise comparisons, we adjusted the probability level by applying a modified Bonferroni correction with the significance threshold set to *p* < 0.025.

**Table 2:** Analyses of Variance (ANOVAs) on the mean amplitudes of the early positivity (P200) and the late sustained negativity.

**Source**		**Early positivity (P200, 150–250 ms)**			**Late sustained negativity (250-800 ms)**		
		**Midline ANOVA**			**Midline ANOVA**		
	**Df**	***F***	***MSE***	***P***	***F***	***MSE***	***P***
Information	(3, 57)	2.35	1.30	0.109	7.70	2.44	0.002^*^
Information^*^Electrodes	(6, 114)	3.99	1.07	0.002^*^	3.77	1.53	0.003^*^
		**Lateral ANOVA**			**Lateral ANOVA**		
	**Df**	***F***	***MSE***	***P***	***F***	***MSE***	***P***
Information	(3, 57)	5.09	0.64	0.008^*^	12.25	0.95	0.001^**^
Information^*^Electrodes	(12, 228)	3.85	1.86	0.002^*^	3.75	2.56	0.002^*^
Information^*^Hemisphere	(3, 57)	6.83	1.93	0.001^*^	5.90	2.65	0.001^*^
Information^*^Electrodes^*^Hemisphere	(12, 228)	1.24	0.35	0.282	0.95	0.51	0.483

Information = Informational Status

* *p < 0.05*,

** *p < 0.001*.

#### 150–250 ms (P200)

Only the lateral ANOVA revealed a main effect of Informational Status, which suggested that the focus-marked target words (both the contrastive and focused conditions) elicited greater positivity than the neutral target word: Contrastive vs. Neutral, *t*_(19)_ = 3.04, *p* < 0.01; Focused vs. Neutral, *t*_(19)_ = 3.81, *p* < 0.005. No other significant differences between conditions were found.

**Figure 2:**
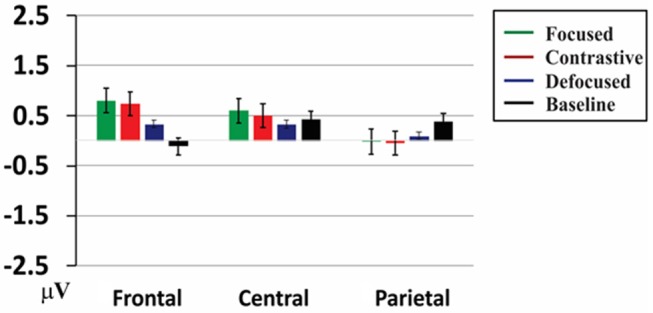
Interaction of the Information × Electrode for the early P200 effect. Mean amplitudes of each condition were averaged across the midline and lateral clusters for each frontal, central, and parietal site from 150–250 ms after the target word onset. The y-axis indicates the amplitude values in μV, positivity upward. Error bars show the standard error of the mean.

Both the midline and lateral ANOVAs indicated significant interaction of Informational Status x Electrodes. Figure 2 highlights the major pattern of the P200 effect varied by the scalp topography. As shown in Figure 2, the focus effect was prominent over the frontal scalp sites in larger P200 relative to both the Defocused and Neutral conditions whereas at the parietal sites the similar focus effect tended to manifest in an opposite manner—the focused target words showed less positivity as compared to the neutral words, suggesting a dipolar pattern of the ERP effect along the frontal-parietal sites. Further resolution of the interaction confirmed this observation. At the anterior (F7-F8 and F3-Fz-F4) sites, the focus-marked words (both the contrastive and focused conditions) elicited larger positivity compared to the neutral target words, Contrastive vs. Neutral: Fz *t*_(19)_ = 3.40, *p* < 0.005, F3-F4 *t*_(19)_ = 3.96, *p* < 0.005, F7-F8 *t*_(19)_ = 2.19, *p* = 0.041; Focused vs. Neutral, Fz *t*_(19)_ = 5.36, *p* < 0.001, F3-F4 *t*_(19)_ = 5.70, *p* < 0.001, F7-F8 *t*_(19)_ = 2.36, *p* < 0.03. No significant differences were found when comparing the Defocused condition with the other conditions, max *t*_(19)_ = 1.875, *p* = 0.076. In contrast, the parietal sites suggested an opposite pattern—both the contrastive and the focused conditions showed relatively less positivity as compared to the neutral condition, Contrastive vs. Neutral, *t*_(19)_ = −2.46, *p* < 0.03, Focused vs. Neutral, *t*_(19)_ = −2.37, *p* < 0.03, consistent with a dipolar ERP pattern. Furthermore, the resolution of the interaction between Informational Status and Hemisphere suggested a somewhat left-lateralized distribution for the P200 effect. The focus-marked words were more positive compared to the neutral words over the left hemispheric scalp sites, Contrastive vs. Neutral, *t*_(19)_ = 4.81, *p* < 0.001; Focused vs. Neutral, *t*_(19)_ = 6.83, *p* < 0.001. No significant differences were found over the right hemisphere scalp sites.

#### 250–800 ms (Central-Frontal Sustained Negativity)

As shown in Table 2, both the midline and lateral analyses suggested a main effect of Informational Status, *Fs* > 7.5, that also interacted with Electrodes, *Fs* > 3.7. The lateral analysis additionally indicated an interaction with Hemisphere. For the main effect of Informational Status, *post-hoc* comparisons suggested two major patterns: first, the Neutral condition elicited more negativity than other conditions—it was more negative than the Contrastive and the Focused conditions in the midline sites, Focused vs. Neutral *t*_(19)_ = 2.68, *p* < 0.03, Contrastive vs. Neutral *t*_(19)_ = 3.07, *p* < 0.01; and the lateral sites, Focused vs. Neutral *t*_(19)_ = 4.56, *p* < 0.001, Contrastive vs. Neutral *t*_(19)_ = 7.08, *p* < 0.001. The Neutral condition also was more negative than the Defocused condition in the lateral sites, *t*_(19)_ = 4.22, *p* < 0.001; second, the Defocused condition, in the midline sites, elicited grater negativity than both the Focused, *t*_(19)_ = 2.50, *p* < 0.03, and the Contrastive conditions, *t*_(19)_ = 3.38, *p* < 0.005, and it pattered with the Neutral condition, *t*_(19)_ = 1.20, *p* = 0.245. No other significant differences were found.

**Figure 3:**
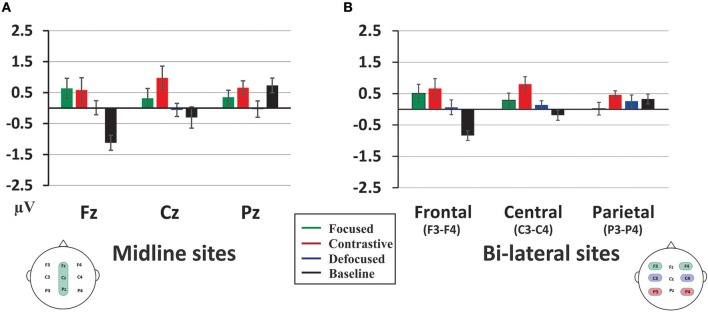
Interaction of the Information × Electrode for the sustained negative ERPs of **(A)** the midline ANOVA and **(B)** the lateral ANOVA. The y-axis indicates the amplitude values in μV, positivity upward. Error bars show the standard error of the mean.

Resolving the interaction of Informational Status × Electrodes indicated that the ERP shifts associated with different focus status varied topographically (Figure 3). The greater negativity induced by the Neutral condition, relative to the other conditions, distributed predominantly at the frontal sites, including both midline Fz [Neutral vs. Contrastive, *t*_(19)_ = 3.06, *p* < 0.01; Neutral vs. Focused, *t*_(19)_ = 4.53, *p* < 0.001; and Neutral vs. Defocused, *t*_(19)_ = 3.59, *p* < 0.005] and bi-lateralized frontal F3-F4 sites [Neutral vs. Contrastive, *t*_(19)_ = 3.81, *p* < 0.005; Neutral vs. Focused, *t*_(19)_ = 4.83, *p* < 0.001; and Neutral vs. Defocused, *t*_(19)_ = 3.06, *p* < 0.01]. For the Contrastive condition, it was less negative-going compared to the other condition, with predominant distribution over both the midline Cz [Contrastive vs. Focused, *t*_(19)_ = 2.43, *p* < 0.03; Contrastive vs. Defocused, *t*_(19)_ = 2.48, *p* < 0.03; and Contrastive vs. Neutral, *t*_(19)_ = 2.30, *p* = 0.033] and bi-lateralized central C3-C4 sites [Contrastive vs. Focused, *t*_(19)_ = 2.65, *p* < 0.03; Contrastive vs. Defocused, *t*_(19)_ = 2.61, *p* < 0.03; and Contrastive vs. Neutral, *t*_(19)_ = 3.75, *p* < 0.005], which also extended to the bi-lateralized parietal sites (P3-P4) but only when comparing with the Focused condition, *t*_(19)_ = 2.82, *p* < 0.03. Additionally, the midline parietal site (Pz) indicated that the Defocused condition was more negative than both the Contrastive, *t*_(19)_ = 2.68, *p* < 0.03, and the Neutral, *t*_(19)_ = 2.55, *p* < 0.03, conditions. No other significant differences were found. Finally, resolving the Informational Status × Hemisphere interaction suggested that the Neutral condition was more negative than the other conditions [Neutral vs. Focused, *t*_(19)_ = 4.03, *p* < 0.005; Neutral vs. Contrastive, *t*_(19)_ = 4.73, *p* < 0.001; and Neutral vs. Defocused, *t*_(19)_ = 5.77, *p* < 0.001] over the left-lateralized sites whereas no significance difference between conditions were found over the right-lateralized sites.

## Discussion

In this event-related brain potential study, we manipulated whether a target word was contextually focused, contrastively focused, contextually defocused, or contextually neutral in a single experiment. This allowed us to examine the effects of different types of focus during online discourse processing. We found that the focus-marked target words (both contrastive and focused) elicited a larger frontal P200 relative to the neutral condition, followed by a central-frontal negative-going waveform that was sustained in the time-course. This sustained negative ERPs showed condition divergence from ~250 ms: Target words that were not focused (i.e., defocused and neutral) elicited increased negativity as compared to target words that were designated focus (i.e., focused and contrastive). Additionally, the sustained negativity showed differences in scalp distribution for different types of focus, suggesting that different neural activities might be involved. The enhanced negativity elicited by the neutral condition, relative to the other conditions, distributed predominantly over the *frontal* and *left*-lateralized sites, whereas the negativity at the *central*-*parietal* sites highlighted a significant reduction in amplitudes of the contrastive condition relative to the other conditions. The enhanced negativity elicited by the defocused condition, relative to both the contrastive and focused conditions, was most pronounced at the midline sites.

We identified the early positivity as a P200 effect given its peak latency and functional associates. The P200 component has been found to reflect attention allocation processes, with a larger P200 associated with attended stimuli relative to unattended stimuli. In our data, the P200 was larger for the focus-marked words (both contrastive and focused) relative to the neutral target words. This suggests that material that was designated focus attracted extra attention, and this attention allocation process was then reflected in the amplitude of the P200, with greater positivity for the focus-marked words compared with the neutral target words. This pattern is in line with previous studies demonstrating an early ERP effect (~200 ms) that was linked to increased attention allocated to the focused materials (Klin et al., 2004; Sanford et al., 2006; Chen et al., 2012). Additional evidence reinforcing this interpretation comes from a recent fMRI study (Kristensen et al., 2013) that reported greater activation of the fronto-parietal attention network when focused information was marked by pitch accent as compared to non-focused information. These results also corroborate the notion (Benatar and Clifton, 2014) that studies that manipulated focus by treating the answers to a *wh*-question as focused, similar to what we did, have consistently demonstrated that focused materials received more attention (Cutler and Fodor, 1979; Blutner and Sommer, 1988).

Following the P200 component, the focus-marked words elicited reduced amplitudes of sustained negative-going waveform relative to the non-focused words. This later ERP effect, in addition, showed graded degrees of focus processing—the more increased focus, the greater reduction in negativity. That the amplitudes of the central-frontal sustained negative ERPs to the neutral target word were greatly enhanced suggests difficulty in integrating this word with the discourse meaning, consistent with our hypothesis. This pattern is also in line with Burkhardt (2006), who reported enhanced N400 to the determiner phrases (e.g., *the conductor*) that were new to the discourse context (i.e., *Tobias talked to Nina*), reflecting costly integration of a new referent to the context meaning. It is, however, unlikely that the observed negativity would be fully accounted for by the typical semantic N400 effect: the central-frontal distribution and the sustained nature of this effect leave open the possibility that it is instead related to the frontal negativity that has been linked to reference-tracking at the level of a situation model (Van Berkum et al., 2007) or to cognitive demands for recruitment of additional memory resources (Ruchkin et al., 1988; Rösler et al., 1995). In the present study, the context and question sentences were packaged to evoke alternatives that license focus marking among multiple possible true candidates in the answer, i.e., the target sentence (Karttunen, 1977; Rooth, 1992). This setup enabled the processing of the target words in the answers to reflect the effect of contextually focused prominence on readers' retrieval of text memory when tracking and updating the reference of the discourse representation. In this context, the temporal and spatial characteristics of ERPs may suggest combined effects in the target-word sustained negativity: an N400 reflecting a meaning integration effect, and a frontally dominant shift reflecting continuing memory and control operations that update and establish referential meaning in discourse. Both of these processes are needed to support the meaning derivation of a referentially-specified meaning of the text (i.e., the situation model). In this view, the reduced amplitudes elicited by the focus-marked words relative to the neutral target words suggest that focusing eased meaning integration during the informationally-based discourse processing. Additionally, the early P200 that was larger for the focus-marked words may suggest that the ease of integrating the focused words with the discourse meaning was promoted by the increased attention paid to the focused items.

Our results, showing that focus facilitates the meaning integration process and that such processing advantage is associated with graded degrees of focus, are in line with previous demonstrations showing that increased linguistic focus is associated with shorter reading times (Morris and Folk, 1998; Birch and Rayner, 2010; Chen et al., 2012), and that referents that are focus-congruent with the information structure of the discourse context elicit smaller negativity than those focus-incongruent ones (Cowles et al., 2007). These results also are consistent with the bulk of evidence demonstrating that focus facilitates language processing in both listening and reading comprehension, which has been thought to reflect the idea that focused information would attract attention more effectively compared to non-focused information (Cutler and Fodor, 1979; Sturt et al., 2004), and that focus would enhance the relative availability of focused concepts in memory (McKoon et al., 1993; Gernsbacher and Jescheniak, 1995). The current results are inconsistent with previous work showing that increased linguistic focus is associated with longer reading times (Birch and Rayner, 1997; Price and Sanford, 2012; Benatar and Clifton, 2014; Lowder and Gordon, 2015). In particular, studies that compared different types of focus processing, similar to what we did in the present study, reported that increased linguistic focus was associated with longer reading times, reflecting costly processing for deeper encoding of the focused word (Lowder and Gordon, 2015) and when the discourse content requires updating/revision of readers' discourse representation (Benatar and Clifton, 2014).

We suggest that the inconsistent effects of focus that have been observed during the real-time processing in the studies with similar approach (comparing different kinds of focus) may be related to the use of different focus-marking devices as well as different linguistic contexts in supporting meaning integration. Our results may differ from these studies because we used the *wh*-question-answer pairs as the focus-marking devices in a discourse context that prompted heightened accessibility of the focused referents. Previous studies have demonstrated that manipulating focus by setting the focus items in response to a preceding *wh*-question would enable the focused materials to receive extra attention (Benatar and Clifton, 2014) and the focused concept to be subject to enhanced priming (Blutner and Sommer, 1988). Our findings, that the focus-marked words, compared with the neutral target words, elicited a larger P200 and reduced negativity of the following sustained ERPs, are in close agreement with these studies (Cutler and Fodor, 1979; Blutner and Sommer, 1988; Chen et al., 2012). This suggests that items highly congruent with the focus-marked prominence designated by the *wh*-question context attracted extra attention, hence eliciting a larger P200, and were more easily integrated with context meaning, hence reduced amplitudes of the negative ERPs, (Cowles et al., 2007). In contrast, previous experimental studies using similar approaches tended to manipulate focus with question-answer pairs of different kinds like the Yes-No questions with *someone/somewhere* in a discourse (Benatar and Clifton, 2014) and with specific syntactic structures like pseudoclefting (e.g., *What the secretary typed was the official memo about the new office policy*) and clefting (e.g., *It was the secretary that typed the official memo about the new office policy*) in a sentence (Lowder and Gordon, 2015). These studies also varied considerably in trial settings. For instance, Benatar and Clifton (2014) examined discourse focus processing with discourses of varying lengths across experiments: e.g., two-sentence discourses in Experiments 1 and 2 and three-sentence discourses in Experiment 3. Lowder and Gordon (2015) examined the processing of syntactic focus in a single sentence. Unlike our use of *wh*-questions as the context to signal the focus-marking in the following answers, the focus manipulations and trial settings used by these studies might not have been able to attract sufficient attention on the focused items, and thus might not have been able to prompt the facilitation observed in our data. We also noted that in our stimuli, the three discourse referents in the meaning representation would be made highly prominent as they were initially introduced by a conjoined NP that was the subject of the initial setup sentence (Gordon et al., 1999). This also contrasts with previous relevant work that, by and large, introduced the target referents with less structural prominence: e.g., the object of a main-verb (Benatar and Clifton, 2014) and adjunct phrase of the initial sentence.

It is likely that focus may have eased discourse integration in the current paradigm, as the candidate referents were made highly accessible in the initial setup sentence and the subsequent *wh*-question-answer device additionally prompted increased attention for deeper encoding of the focused item. The focused referent, therefore, becomes highly accessible and is more easily integrated to form a coherent linguistic (propositional) representation of the text in memory. The integration would also likely enhance the prominence of the focused referent in that representation (Gernsbacher and Jescheniak, 1995), which in turn may result in increased activation of the items that are semantically and propositionally related to the focused referents in text memory, reflecting in reduced negativity on the target word when it was congruent with the focus-marked prominence and when it was semantically related to the focused concept. In support of this idea, Morris and Folk (1998) manipulated focus in context and reported faster reading times on the *subsequent* target word that was semantically related to the previously focused concept as compared to the non-focused concept. Morris and Folk (1998) attributed the processing advantage of focused over non-focused conditions in measures on the subsequent target words to reflect the message-level context effects in sentences—the influence of properties of a discourse/text like causal relations (Suh and Trabasso, 1993), focus (Grosz et al., 1983; Garrod and Sanford, 1990), discourse topic (Chafe, 1976), etc. on the processing of subsequent individual words during reading. Binder and Morris (1995), in addition, showed that items related to the topic of the discourse were more easily integrated into the discourse representation than unrelated items. On this basis, Morris and Folk (1998) interpreted the facilitation as evidence that focus would be an effective message-level contextual priming mechanism that promotes the access to semantically related items in the lexicon during reading. More specifically, the message-level priming in a discourse could be propositionally-based (Ratcliff and McKoon, 1978; Long et al., 2005), and thus the focused referent that calls for an updating of a discourse model (contrastive) might not be as costly as would be needed given that referents relevant to the propositional content of the text were primed to be readily accessible in text memory.

While we have interpreted the later negative ERPs to reflect the semantic and referential processes that integrate the word's meaning with the meaning of the text, one important question is whether such ERP responses may be affected by a lower-level lexical association, reflecting lexical priming like item repetition. For instance, the information structure has been typically divided into two complementary parts, like focus vs. background or new vs. given, and our manipulation of Focused and Defocused resembles the division of new vs. given in the information structure. It has been shown that information that was new (also focused information) to the discourse induced more costly processing than information that was already mentioned in the discourse (i.e., the given information), reflected in greater amplitudes of the N400 to new referents as compared to given referents (Burkhardt, 2006) and in longer reading times for focus items relative to given items (Benatar and Clifton, 2014). If lexical association accounts for the observed effects, then we would expect to see reduced negativity in the defocused condition relative to the focused condition because the defocused target words, in the context of *who*-question, would be made given (Burkhardt, 2006). This pattern, however, did not occur; instead, the defocused target word (i.e., given information) demonstrated enhanced negativity compared to both the focused and the contrastive conditions. This pattern, on the other hand, is consistent with Birch and Rayner (2010), who showed longer reading times for the non-focused materials relative to the focused materials, and Cowles et al. (2007), who showed greater N400 for the focus-incongruent words than for the focus-congruent words. These results suggest the prominent influence of the focus effects based primarily on the information-structural constraints. One may note that the defocused condition, as shown in Figure 1 (i.e., Cz), might seem similar to the focused condition in an earlier time window of the sustained negative ERPs, suggesting a possible lexical priming that was weaker than would have been expected; however, supplementary analysis indicated that this effect was not reliable (see Footnote 2). Similar pattern of the defocused condition was also shown in the P200 waveform. As shown in Fz of Figure 1, the defocused condition in the 150–250 ms time window appeared to transiently pattern with the focused condition and then deflected more toward the neutral condition. There was no significance when comparing the defocused condition with the focus-marked conditions and with the neutral condition, suggesting that the effect was transient and highly volatile. Thus, lexical priming may transiently affect the ERPs observed in our data, but it will not necessarily do so. There is an effect of informationally based mechanisms. This conclusion corroborates other research showing that the meaning integration processing is affected by factors [e.g., message (context/discourse) level, information structure] beyond specific lexical factors (Ledoux et al., 2006).

Another important question is whether the notable discrepancy between the behavioral results and the ERP results in our data may undermine our interpretation of the results: We found focus effect in the ERP results (i.e., the early P200 and the late negative ERPs) not the behavioral results (participants' response times to the comprehension questions). It is noteworthy that such discrepancy reflects the distinction between the online and offline tasks. Online tasks like the ERP method and eye-tracking method can track processing of the comprehension as it temporally *unfolds*. Online measures reflect the detailed timecourse of mental activation of different kinds of lingusitic information when the integration processing occurs whereas offline measures like the response times to the comprehension questions in our data reflect processing loads related to the *results* of comprehension. They contribute to the generation of meaning representation for the comprehension at different levels of characteristics. Our results that mainly online measures (i.e., ERPs) showed sensitivity to the focus manipulations suggested that focus exerted an effect primarily on the temporal and logical flow of information when the meaning integration processing occurs, but much less on the later elaborate processing that tapped into the knowledge representation stored in the reader's long-term memory. This conclusion is in agreement with previous studies reporting focus effect mainly in online measures (e.g., ERPs: Cowles et al., 2007; Chen et al., 2014, Eye-tracking reading times: Chen et al., 2012; Benatar and Clifton, 2014; Lowder and Gordon, 2015).

To summarize, the present study adds to the previous research showing focus effects in reading integration processing in the following specific ways. First, with the inclusion of a *range* of focus conditions, our results show that increased focus information would ease the integration of the focused word with the preceding context meaning. In particular, our results suggest that focusing a word in a discourse context would function to attract additional attention on the focus-marked material and promote the prominence of the focus-marked items. The prominence of focused referents would likely prompt the message-level contextual priming that facilitates the access to referents that are propositionally relevant. Furthermore, the effect we observed shows scalp distribution differences varied by the focus types. Previous ERP work suggested that ERPs recorded in the same time window with different topography might index different processes (Dien et al., 2010). For instance, Dien and O'Hara (2008) reported that the effect of semantic priming was distributed predominantly over the left parietal sites, whereas Franklin et al. (2007) reported that the effect of semantic matching was distributed predominantly over the right parietal sites. In our data, the differential topography for different types of focus processing may reflect differential activation of neural substrates, suggesting that different cognitive processes may be involved for processing different types of focus. Although we cannot infer the cortical sources of these differences, it is noteworthy that a medial prefrontal cortex source localized to the Nref (Nieuwland et al., 2007b) would be consistent with the major topographical pattern of ERP effects in our data, which were distributed predominantly over the frontal and central sites. This suggestion also lends support to the theoretical framework that distinguishes the focus concept into three types of linguistic functions and representations: i.e., contrastive focus, discourse-newness (informational focus) and givenness (Féry and Samek-Lodovici, 2006; Selkirk, 2007).

Note that we do not claim that the differential ERP topography sensitive to our focus manipulation indicates that different types of focus would be processed and represented distinctively. Although our ERP evidence demonstrates topographical differences between contrastive and informational focus, suggesting at least functional differences among them, the stronger interpretation, that contrastive and informational focus are processed independently, has yet to be determined empirically. The theoretical question of interest, then, is whether contrastive focus would be represented independently from informational focus in our linguistic knowledge (Halliday, 1967; Kiss, 1998; Katz and Selkirk, 2011), or if it belongs to a similar representation that carries certain specific functional features during the operation of informationally-based processes (e.g., the “exhaustiveness” in Torregrossa, 2012). Clearly, more research is needed to disentangle the nature of processing/representational differences between contrastive focus and informational focus. In any case, the current results suggest that different types of focus, at least for the type of question-answer manipulations in our investigation, have functional consequences in the semantic and referential processes of meaning integration.

That focus has been presented as a broad, multifaceted construct has made it likely that the investigation of the cognitive mechanisms underlying the online processing of linguistic focus would be susceptible to the particular type of manipulation in the experiment (Benatar and Clifton, 2014; Lowder and Gordon, 2015). Be that as it may, we have demonstrated that by setting the referents with high prominence in the text representation and using *wh*-question-answer pairs as the focus-marking devices embedded in a context of sufficient length, we see an obvious facilitation of meaning integration on the processing of the target word when it is congruent with the focus-marking. This leads us to suggest that the mechanisms that support the integration of the focused item to the context meaning in a discourse may be susceptible to the prominence of candidate referents for the target focused constituent, the build-up of context support, and the use of focus-marking devices. We echo Lowder and Gordon (2015) suggestion that it is important to consider a *range* of focus conditions in investigating the nature of focus effects in the real-time language processing, as such an approach would provide fine-grained information to disentangle the relative prominence and interactive dynamics among different types of focus processing.

## Author Contributions

CLY designed and conducted the experiment, analyzed the data, determined the results presentation and interpretation, and was the primary writer of the manuscript. HZ also contributed to the experiment design and material refinement. HP and HZ jointly determined the results interpretation and were the secondary drafter of the manuscript. HD contributed to the stimuli refinement.

### Conflict of Interest Statement

The authors declare that the research was conducted in the absence of any commercial or financial relationships that could be construed as a potential conflict of interest.

## Footnotes

^1^Van Berkum et al. (1999) presented participants mini-stories where the number of candidate referents for the target definite noun phrase was manipulated in the context sentences, shown below (*the girl* in the final sentence is the target phrase where the ERPs were extracted and examined). (1a) *David had asked the boy and the girl to clean up their room before lunchtime. But the boy had stayed in bed all morning, and the girl had been on the phone all the time. David told* the girl *that had been on the phone to hang up*. (1b) *David had asked the two girls to clean up their room before lunchtime. But one of the girls had stayed in bed all morning, and the other had been on the phone all the time. David told* the girl *that had been on the phone to hang up*. Note that for the target phrase *the girl* (underlined) in the final sentence, the discourse context provided a single unique referent in (1a), but two equally eligible referents in (1b). Thus, the target phrase *the girl* was referential ambiguous in (1b) but referentially unambiguous in (1a). The results indicated that, relative to *the girl* in (1a), *the girl* in (1b) elicited sustained, negative-going deflection, emerging at about 280 ms after the noun onset and with maximal distribution at the anterior sites. The timecourse and topographic distribution of the extended negative ERP effect suggests qualitatively difference from the ERP effect reflecting the analysis of syntactic structure (i.e., the P600) and the integration of word meanings (i.e., the N400).

^2^To enable comparisons with previous work, we also conducted statistical tests on the mean amplitudes of separate time windows: 250–500 and 500–800 ms. The results were largely the same to what we did in the present study (250–800 ms please refer to Appendix); crucially, no qualitative differences involved among condition comparisons. To illustrate, the results of both time windows showed the same pattern of defocused effects in the midline analyses—the Defocused condition was more negative than both the Focused condition: 250–500 ms, *t*_(19)_ = 2.28, *p* < 0.04, 500–800 ms, *t*_(19)_ = 2.66, *p* < 0.03, and the Contrastive condition, 250–500 ms, *t*_(19)_ = 3.08, *p* < 0.01, 500–800 ms, *t*_(19)_ = 3.24, *p* < 0.005, despite the observation in Figure 1 (Cz) that the defocused condition seemed to behave like the focused condition in an earlier 250–500 ms time window. In addition, the patterning between the Defocused and the Neutral conditions was confirmed in the midline analyses of both time windows: 250–500 ms, *t*_(19)_ = 1.80, *p* = 0.090, 500–800 ms, *t*_(19)_ = 0.74, *p* = 0.470.
