# Supplementary material for: Linguistic Focus Promotes the Ease of Discourse Integration Processes in Reading Comprehension: Evidence From Event-Related Potentials
Source: Front Psychol. 2019 Feb 1;9:2718. doi: 10.3389/fpsyg.2018.02718 (PMC6367260; doi:10.3389/fpsyg.2018.02718)
Supplement: Supplementary file 1 [file Table_1.docx]

## Appendix

We defined the time windows in view of the effects revealed by visual inspection of the grand average ERPs and on the basis of previous studies (Chen et al., 2014; Cowles et al., 2007). The visual inspection suggested a steady negative shift, which showed a major divergence among conditions from ~250 ms (Cz) that remained largely similar over a sustained time-course. We tested this ERP effect with separate time windows 250-500 ms and 500-800 ms (Chen et al, 2014; Cowles et al., 2007) which yielded similar experimental effects. The following Table highlights the similarities of the results of the two separate time windows.

Table: Analyses of Variance (ANOVAs) on the mean amplitudes of the time windows 250-500 ms and 500-800 ms.

| **Source** |  | **250 to 500 ms** | | | | **500 to 800 ms** | | |
| --- | --- | --- | --- | --- | --- | --- | --- | --- |
|  |  | ***Midline ANOVA*** | | | | ***Midline ANOVA*** | | |
|  | **Df** | *F MSE* | | *P* | *F MSE* | | | *P* |
| **Information** | (3, 57) | 8.15 | 2.57 | 0.002* | 6.29 | | 3.10 | 0.006* |
| **Information*Electrodes** | (6, 114) | 4.21 | 1.91 | 0.004* | 3.18 | | 2.34 | 0.016* |
|  |  | ***Lateral ANOVA*** | | | | ***Lateral ANOVA*** | | |
|  | **Df** | *F MSE* | | *P* | *F MSE* | | | *P* |
| **Information** | (3, 57) | 11.20 | 1.09 | 0.001** | 11.34 | | 1.24 | 0.001** |
| **Information*Electrodes** | (12, 228) | 4.56 | 3.33 | 0.002* | 2.97 | | 3.94 | 0.019* |
| **Information*Hemisphere** | (3, 57) | 4.32 | 2.71 | 0.010* | 5.98 | | 4.23 | 0.003* |
| **Information*Electrodes*Hemisphere** | (12, 228) | 1.30 | 0.76 | 0.269 | 0.83 | | 0.83 | 0.547 |

That the sustained nature of this central-frontal negative effect and the mirrored results of the two time windows suggests that our experimental manipulation yielded a relatively extended ERP effect, similar to frontal effects that were linked to the processing of referential and semantic ambiguity (Lee & Federmeier, 2009; Van Berkum et al., 2007; Yang et al., 2010). In these studies, extended time windows have been used to assess the ERP effect. In this context, we adopted an extended time window that seems to be more appropriate to assess the extended ERP effect.
